# A Waypoint Tracking Controller for Autonomous Road Vehicles Using ROS Framework

**DOI:** 10.3390/s20144062

**Published:** 2020-07-21

**Authors:** Rodrigo Gutiérrez, Elena López-Guillén, Luis M. Bergasa, Rafael Barea, Óscar Pérez, Carlos Gómez-Huélamo, Felipe Arango, Javier del Egido, Joaquín López-Fernández

**Affiliations:** 1 Electronics Department, University of Alcalá, Campus Universitario, 28805 Alcalá de Henares, Spain; rodrigo.gutierrez@edu.uah.es (R.G.); luism.bergasa@uah.es (L.M.B.); rafael.barea@uah.es (R.B.); o.perezg@edu.uah.es (Ó.P.); carlos.gomezh@edu.uah.es (C.G.-H.); juanfelipe.arango@edu.uah.es (F.A.); javier.egido@edu.uah.es (J.d.E.); 2 Systems Engineering and Automation Department, University of Vigo, 36310 Vigo, Spain; joaquin@uvigo.es

**Keywords:** path tracking control, autonomous road vehicles, Robot Operating System (ROS)

## Abstract

Automated Driving Systems (ADSs) require robust and scalable control systems in order to achieve a safe, efficient and comfortable driving experience. Most global planners for autonomous vehicles provide as output a sequence of waypoints to be followed. This paper proposes a modular and scalable waypoint tracking controller for Robot Operating System (ROS)-based autonomous guided vehicles. The proposed controller performs a smooth interpolation of the waypoints and uses optimal control techniques to ensure robust trajectory tracking even at high speeds in urban environments (up to 50 km/h). The delays in the localization system and actuators are compensated in the control loop to stabilize the system. Forward velocity is adapted to path characteristics using a velocity profiler. The controller has been implemented as an ROS package providing scalability and exportability to the system in order to be used with a wide variety of simulators and real vehicles. We show the results of this controller using the novel and hyper realistic CARLA Simulator and carrying out a comparison with other standard and state-of-art trajectory tracking controllers.

## 1. Introduction

In recent years, both the research community and companies have paid special attention to the topic of autonomous driving. Automated Driving Systems (ADSs) have the potential to radically improve transport and mobility by enhancing the safety and efficiency of road vehicles. A good part of these research efforts focuses on perception and interpretation of the complex and dynamic environment that includes other vehicles, traffic signaling, urban infrastructure and pedestrians [1,2]. However, these efforts would be useless without a robust planning and control system that allows a safe and fast execution of the planned movements, through convenient feedback control loops.

The great computation power of current onboard processors has leveraged research in machine and deep learning methods in all ADS modules, including planning and control [3,4]. The advantage of these control methods is their ability to drive in the absence of maps, relying on a comprehensive understanding of the surrounding environment while following high level learnt commands. However, these methods require arduous and specific training for each environment, and the results in the field of trajectory tracking control are not yet robust or reliable.

On the other hand, most approaches to date focus on navigation architectures that define driving behavior in annotated 3D geometric maps [5,6,7]. Currently, there are a wide variety of companies [8,9] and open source applications such as OpenStreetMap [10] or OpenDRIVE [11], that provide tools to drastically reduce the time and effort required to create these maps, and these are in continuous development due to the rise of GPS-based positioning systems.

**Figure 1:**
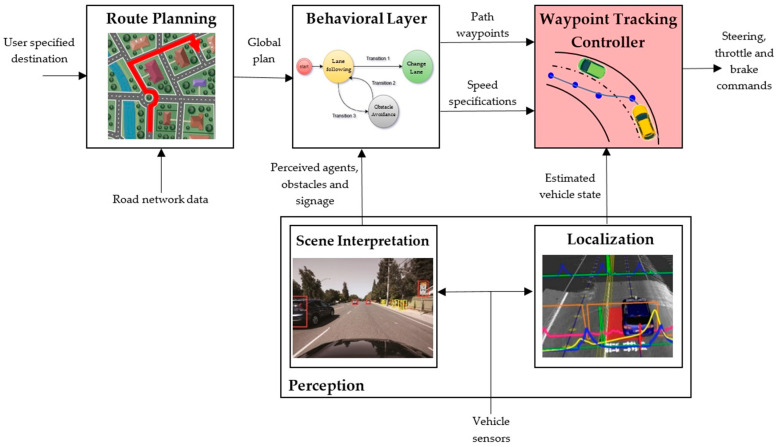
General navigation architecture of an autonomous vehicle. This work proposes a solution for the Waypoint Tracking Controller that receives as inputs a set of path waypoints and speed specifications along with the estimated vehicle state and generates as outputs the vehicle control signals.

With either of the two previous approaches, and although other alternatives to recreate the horizontal alignment of a road have also been proposed [12], most global planning systems provide as output a set of waypoints to follow to complete the planned route [13]. Figure 1 shows a typical navigation and decision-making architecture for which the proposed tracking controller provides a robust solution in the motion and feedback control layer. From user specifications and road network data, the route planning system elaborates a global plan. A stream of observations from on-board sensors such as LIDARs, cameras, GPS/INS units and odometry is processed by the scene interpretation module in order to provide knowledge about the environment of the vehicle, the perceived agents, obstacles or traffic signaling. The behavioral layer is responsible for selecting an appropriate driving and motion behavior at any point of time based on the information of the scene interpretation module. When the behavioral layer decides on the driving behavior to be performed in the current context (e.g., lane keeping, lane changing or right turning), the selected behavior is translated into a path or trajectory that must be tracked by the low-level tracking controller. A convenient and usual way to define this path is to generate a sequence of future waypoints the vehicle has to go through. Finally, a feedback tracking controller is needed to select the appropriate actuator inputs to follow the planned waypoints also using the estimated vehicle pose and additional speed specifications from the behavioral layer. In this work, a robust and real time solution for this waypoint tracking controller is proposed, providing an open-source implementation as a package for the Robot Operating System (ROS) environment [14].

Waypoint tracking controllers must consider feasibility, comfort and vehicle dynamics in order to follow a trajectory. Interpolation techniques take the set of global waypoints and generate a smoother path in benefit of the trajectory continuity and vehicle constraints. In the field of automated driving, different solutions have been proposed [13,15,16]. A very simple mathematical solution for car-like robots is to join waypoints with straight and circular shapes [17,18]. Clothoids have been implemented in the design of highways and are also suitable for car-like robots [19,20] to obtain smooth transitions between straight segments and curved ones. Polynomial curves [21,22] have also been used to meet the constraints needed in the waypoints, such as fitting position, angle and curvature constraints, among others. Another solution with a low computational cost is the Bézier curves [23,24], that rely on control points to define their shape. However, spline curves [25,26,27,28] have several advantages in the field of autonomous driving, due to their very low computational cost and their high degree of smoothness constraint at the joint between the segments of the generated path.

Once the path is interpolated, different tracking controllers can be implemented. The most popular are the kinematic or geometric ones, due to their simplicity and stability. Among them, the most typical implementations are the “follow the carrot” [29], “Pure Pursuit” [30] and “Stanley” [31] controllers. “Pure Pursuit” is by far the most popular among geometric methods, so it has been a standard benchmark to validate new controllers proposed by researchers [32]. The main shortcoming of “Pure Pursuit” is the selection of the “look-ahead distance” parameter, which is very difficult to achieve due to it obeying to a compromise between stability and tracking performance, making this strategy course-dependent. Dynamic controllers [33,34,35] include the dynamic properties of the vehicles in the control law, but need dynamic feedbacks such as force and torque which require expensive dedicated sensors. When high robustness to tracking disturbances is required, a good choice is using adaptive and intelligent control [36,37], although these methods usually take a lot of computational effort. On the other extreme, classical controllers [38,39] can be easily applied to steering actuation, but their adjustment may need complex derivations and selections. Optimal control [40,41] has demonstrated to be a suitable choice for robust applications and allows a very intuitive adjustment of its parameters through the use of cost functions.

Despite the variety of control solutions existing in the literature, there are not yet open-source implementations for waypoint tracking that are scalable, robust and real-time for different kinds of autonomous vehicles or robotic simulators. Robot Operating System (ROS) [14] is a very common programming environment within the robotics research community, that aims to get this scalability and reusability. However, the navigation stack of ROS [42] does not include a solution for waypoints following. Other packages for ROS, such as “follow_wayoints” [43] or “yocs_waypoints_navi” [44], simply buffer the waypoints and manage them as independent goals, without imposing continuity or smoothness constraints to the resultant trajectory.

The objective of this work is to provide a robust, scalable and real-time open-source implementation for a waypoint tracking controller for urban autonomous vehicles as a package of ROS, publicly available through the following GitHub: “Waypoint_Tracking_Controller” [45]. To ensure real-time capabilities, a spline interpolator that meets continuity and smoothness restrictions has been chosen, with an optimal Linear Quadratic Regulator (LQR) lateral control that allows an intuitive and fast parameter adjustment. A velocity profiler optimizes the linear velocity as a function of the trajectory parameters. In addition, a delay compensator minimizes the effect of sensor and actuator delays in the stability of the control loop. The ROS package has been validated using the novel and hyper realistic CARLA Simulator [46], an open-source simulator for autonomous driving research distributed under MIT License. A comparison with “Pure Pursuit” standard and our previous low level controller [47], taken as our baseline in this paper, is carried out in CARLA, showing the good performance of our proposal.

The paper is organized as follows. Section 2 describes the controller design, including vehicle model, interpolation of waypoints, lateral control, linear speed generation and delay compensation. The ROS package implementation is explained in Section 3. Section 4 shows the experimental results and discussion. Finally, in Section 5 the main conclusions of this work are presented.

## 2. Controller Design

The trajectory tracking control system of an autonomous robot is composed of longitudinal and lateral controllers. The longitudinal controller is responsible for regulating the vehicle’s cruise velocity (or linear speed) while the lateral controller steers the vehicle’s wheels for path tracking. If the path is given by a set of waypoints, a trajectory interpolator is necessary to obtain smooth and optimized movements and continuous references for the actuators. An additional challenge in urban autonomous vehicles that can reach high speeds is dealing with the delays introduced by sensors and actuators in the control loop, that can greatly destabilize the system.

In this work, controller design choices have been made taking into account the desired scalability and reusability of the system. The goal is to get an ROS implementation of the controller that can be used by different vehicles and simulators without the need of complex model definitions or parameter settings, and that can be executed in real time with low cost computational resources.

**Figure 2:**
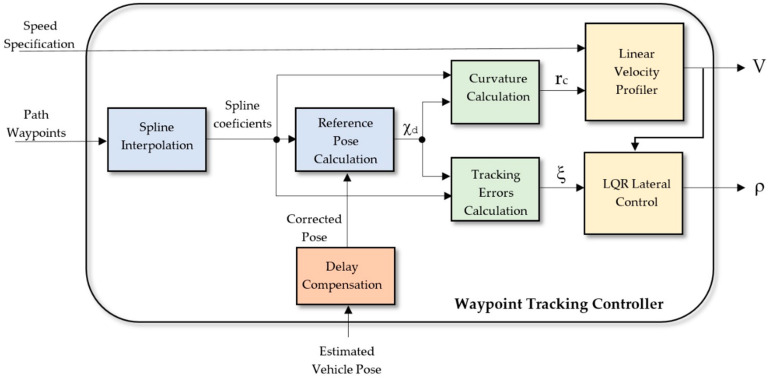
Global architecture of the proposed Waypoint Tracking Controller. Inputs are: a set of path waypoints, an external speed specification and the estimated vehicle pose. Outputs are: linear speed V and steering angle ρ. The main subsystems are a Spline Interpolator, a Linear Velocity Profiler, a Linear Quadratic Regulator (LQR) Lateral Controller and a Delay Compensation System.

The architecture of the proposed controller is shown in Figure 2. Inputs are a set of waypoints and an external speed specification provided by the high level planner, and the estimated vehicle pose obtained from its sensors. A spline interpolator calculates a smooth trajectory, whose curvature at each point is the main input for a linear velocity profiler that adjust the longitudinal velocity of the vehicle V. The lateral control is an optimal LQR controller that uses the tracking errors to steer the vehicle. A delay compensation system offsets the delays introduced by the positioning system of the vehicle and the actuators. The following subsections explain the different modules of the controller.

### 2.1. Vehicle Model and Limitations

The kinematic model of a vehicle assumes it has negligible inertia, and this assumption is effective for typical urban driving speeds [48]. On the other hand, safe autonomous vehicles require speeds that minimize wheel slippage. In this work we use a kinematic model instead a more complex dynamic model because we aim to implement a generic and safe urban autonomous vehicle with limited speed (up to 50 km/h), and we use a feedback control loop for waypoint tracking which absorbs inaccuracies due to the model.

**Figure 3:**
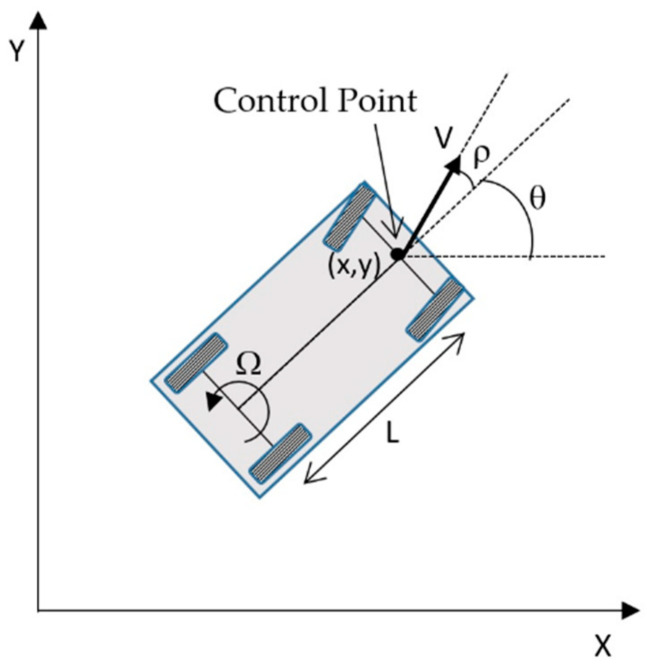
Kinematic model of an automobile with Ackerman steering. The control point is the central point of the front wheel’s axle. The kinematic model obtains the evolution of the location of the control point (*x, y, θ*) as a function of the speed *V*(*t*) and the angle *ρ*(*t*) of a virtual central front wheel at the control point.

To simplify the control of an automobile with Ackerman steering, the following criteria are applied: vehicle pose (*x, y, θ*) is taken with respect to an external reference frame and it is composed of the cartesian coordinates (*x, y*) of the central point of the front wheels axle (control point in Figure 3) and the heading angle *θ* between its longitudinal axle and the external X-axis. The kinematic model allows calculating the evolution of this pose from the speed *V*(*t*) and the angle *ρ*(*t*) of a virtual central front wheel at the control point:

(1) $$\begin{array}{l} \dot{x}\left(t\right)=V\left(t\right)\cdot \cos\left(\rho \left(t\right)+\theta \left(t\right)\right)\\ \dot{y}\left(t\right)=V\left(t\right)\cdot \sin\left(\rho \left(t\right)+\theta \left(t\right)\right)\\ \dot{\theta }\left(t\right)=\Omega \left(t\right)=\frac{V\left(t\right)\cdot \sin\left(\rho \left(t\right)\right)}{L} \end{array}$$

where Ω(*t*) is the angular velocity (first derivative of the heading angle), *L* is the distance between the front and rear axles of the vehicle and the steering is mechanically limited to $\left|\rho \left(t\right)\right|<\rho_{max}$.

### 2.2. Waypoints Interpolation

As it can be seen in Figure 1, the tracking controller receives as input a set of path waypoints to be followed. Some widely used path tracking methods, such as “Pure Pursuit” [30], directly use the next waypoint in the list to implement lateral control, deriving in trajectories that are highly dependent on the distance between waypoints and with discontinuities in speed and acceleration. For driving at high speeds, a smooth path is necessary, and different interpolation methods provide this functionality. Spline interpolation [49,50] have important advantages in terms of computational efficiency, accuracy and smoothness, because it minimizes the curvature of the path, providing a trajectory that exactly pass through input waypoints. For autonomous driving, two-dimensional cubic splines ensure continuity in first and second derivatives, which allows smooth angular and lineal velocities at the joint points.

**Figure 4:**
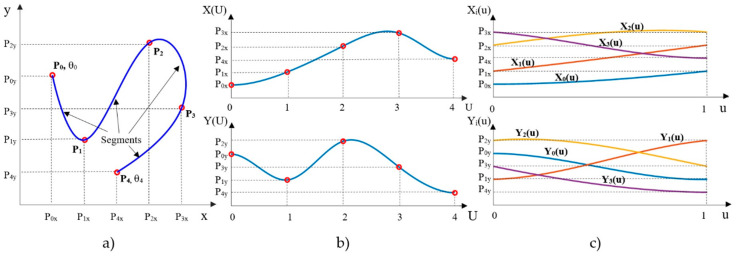
Cubic spline definition: (**a**) Cubic spline in cartesian space x-y, with five waypoints (orientation is imposed at start and goal points) and four segments, (**b**) parametric cubic splines for each coordinate of Cartesian space, X(U) and Y(U), (**c**) polynomials of each segment of the spline with normalized parameter u.

A two-dimensional parametric cubic spline *Q*(*U*) is got by combining two splines, *X*(*U*) and *Y*(*U*), where *U* is the parameter along the curve. Each spline consist of n segments (cubic polynomials), being *n*+1 the number of waypoints *P_i_* (*i* ∈ [0,*n*]) to join (see Figure 4a,b). To simplify segments handling, it is usual to normalize *U* into *u* ∈ [0,1] for each segment, obtaining the spline definition *Q*(*u*) = (*X_i_*(*u*), *Y_i_*(*u*)) ∀ *i* ∈ [0,*n*−1] that is shown in Figure 4c, with cubic polynomials for each segment:

(2) $$\begin{array}{c} X_{i}\left(u\right)=a_{ix}+b_{ix}\cdot u+c_{ix}\cdot u^{2}+d_{ix}\cdot u^{3}0\leq u\leq 1\\ Y_{i}\left(u\right)=a_{iy}+b_{iy}\cdot u+c_{iy}\cdot u^{2}+d_{iy}\cdot u^{3}0\leq u\leq 1 \end{array}$$

The four coefficients of each of the two polynomials *X_i_*(*u*) and *Y_i_*(*u*) of the segment *i* are calculated to fulfill continuity conditions in first and second derivatives at waypoints *P_i_* and *P*_*i*+1_. For the initial and final segments, a certain orientation in the *x-y* plane is also imposed at the initial and final waypoints of the spline: *θ_0_* and *θ_n_*, respectively (see Figure 4a).

For the *Y_i_*(*u*) polynomial of segment *i*, and calling *D_iy_* and *D*_(*i*+1)*y*_ to its first derivatives at points *P_i_* and *P*_*i*+1_, respectively (so *D_iy_ = Y_i_*′(0) and *D*_(*i*+1)*y*_ = *Y_i_*′(1)), we obtain the following expressions for its coefficients:

(3) $$\begin{array}{c} a_{iy}=P_{iy}\\ b_{iy}=D_{iy}\\ c_{iy}=3\left(P_{\left(i+1\right)y}-P_{iy}\right)-2D_{iy}-D_{\left(i+1\right)y}\\ d_{iy}=2\left(P_{iy}-P_{\left(i+1\right)y}\right)+D_{iy}+D_{\left(i+1\right)y} \end{array}$$

To ensure continuity in the second derivative at the intermediate waypoints of the spline (${Y^{\prime \prime }}_{i-1}\left(1\right)={Y^{\prime \prime }}_{i}\left(0\right)$), the following condition must be satisfied:

(4) $$2c_{\left(i-1\right)y}+6d_{\left(i-1\right)y}=2c_{iy}$$

and substituting the coefficients of Equation (3), we obtain:

(5) $$D_{\left(i-1\right)y}+4D_{iy}+D_{\left(i+1\right)y}=3\left(P_{\left(i+1\right)y}-P_{\left(i-1\right)y}\right)$$

The orientation in the x–y plane of the spline in the initial and final waypoints (*θ_0_* and *θ_n_*) can be translated into slope conditions for *Y*(*u*) and *X*(*u*) polynomials in *P_0_* and *P_n_* in the following way:

(6) $$tg\left(\theta_{i}\right)={\frac{dy}{dx}|}_{P_{i}}={\frac{Y^{\prime }\left(u\right)}{X^{\prime }\left(u\right)}|}_{P_{i}}=\frac{D_{iy}}{D_{ix}}=\frac{\mu \cdot sin\left(\theta_{i}\right)}{\mu \cdot cos\left(\theta_{i}\right)}$$

where the parameter μ has impact on the permanence of the imposed orientation in the Cartesian trajectory. Higher values of μ lead to smoother trajectories that more slowly change the orientation, but if the waypoints are very close together, it can lead into more oscillating paths.

Thereby, we obtain a system of (n + 1) equations to obtain the derivatives *D_i_*:

(7) $$\left[\begin{array}{ccccccc} 1 & & & & & & \\ 1 & 4 & 1 & & & & \\ & 1 & 4 & 1 & & & \\ & & 1 & 4 & 1 & & \\ & & & . & . & . & \\ & & & & 1 & 4 & 1\\ & & & & & & 1 \end{array}\right]\cdot \left[\begin{array}{c} D_{0}\\ D_{1}\\ D_{2}\\ .\\ .\\ .\\ D_{n} \end{array}\right]=\left[\begin{array}{c} \mu \cdot sin\left(\theta_{0}\right)\\ 3\left(P_{2y}-P_{0y}\right)\\ .\\ .\\ .\\ 3\left(P_{ny}-P_{\left(n-2\right)y}\right)\\ \mu \cdot sin\left(\theta_{n}\right) \end{array}\right]$$

Once the first derivatives are known for all points, Equation (3) is applied to each segment to obtain their coefficients. The same Equations (3)–(7) can be applied to obtain the coefficients of *X_i_*(*u*) polynomial of segment *i*, replacing sine with cosine functions in Equation (7).

The output of the spline interpolator is the set of polynomial coefficients for each segment of the path. Unlike other approaches using spline interpolation [31,51], we do not obtain a larger sequence of points, which require high computational resources and execution times for the controller to work with them. We work in a parametric way and we only need to store a few polynomial coefficients because the controller has been formulated in terms of these coefficients, as it will be seen in the next subsections.

### 2.3. Linear Velocity Profiler and Curvature Calculation

There are multiple external factors that condition the speed at which the vehicle must drive, such as traffic conditions, signs, road speed limits, etc. These elements must be interpreted from the perception systems and the behavioral layer of the navigation system and translated into certain speed specifications for the control layer. Regardless of these external factors, which are out of the scope of this article, the characteristics of the trajectory itself determine the optimal speed at which the vehicle must travel. From all of them, the most relevant is the curvature *C*, that limits the maximum speed to assure that the vehicle follows the prescribed path minimizing sliding.

When dealing with two-dimensional spline curves, the radius of curvature *rc* (defined as the inverse of the curvature *C*) in the point corresponding to a certain value of the parameter *u* is:

(8) $$rc\left(u\right)=\frac{{\left(X^{\prime }{\left(u\right)}^{2}+Y^{\prime }{\left(u\right)}^{2}\right)}^{3/2}}{X^{\prime }\left(u\right)\cdot Y^{\prime \prime }\left(u\right)-Y^{\prime }\left(u\right)\cdot X^{\prime \prime }\left(u\right)}$$

The linear velocity of an autonomous vehicle can be adjusted as a function of the current radius of curvature [33,38]. However, for road vehicles, it is necessary to reduce speed in advance, before reaching the segments of higher curvature. To achieve this, the velocity profiler first calculates the mean radius of curvature of each segment *i* of the spline (${\bar{rc}}_{i}$). Then, an average speed ${\bar{v}}_{i}$ is assigned to each segment of the spline, which is a function of its average radius of curvature:

(9) $${\bar{v}}_{i}=V_{MAX}\cdot \frac{min\left({\bar{rc}}_{i},\;RC_{MAX}\right)}{RC_{MAX}}$$

where *V_MAX_* is the maximum allowed linear velocity for the vehicle and *RC_MAX_* is the maximum value of the radius of curvature that reduces the linear velocity below *V_MAX_*.

The speed *V_i_* assigned to each segment of the spline is calculated by averaging the *N* subsequent segments velocities, using a vector *λ* of normalized weights:

(10) $$V_{i}=\overset{N}{\underset{n=1}{\sum }}{\bar{v}}_{i+n-1}\cdot \lambda \left(n\right)$$

Finally, a linear interpolator smooths the speed transitions between segments, calculating the velocity command assigned to a value of the parameter *u* within the segment *i* of the spline in the following way:

(11) $$V=V_{i}\left(u\right)=\{\begin{array}{ll} V_{i}+\left(u-0.5\right)\cdot \left(V_{i+1}-V_{i}\right) & 0.5\leq u\leq 1\\ V_{i-1}+\left(u+0.5\right)\cdot \left(V_{i}-V_{i-1}\right) & 0\leq u<0.5 \end{array}$$

### 2.4. Lateral Control

Once the trajectory and linear velocity profile have been defined, a lateral controller provides closed-loop path tracking. This section proceeds by determining a partial control law, assuming the real position of the vehicle is known (“corrected pose” in Figure 2) and the vehicle moves at the linear velocity V calculated by the “Linear Velocity Profiler”. In next section, the control law will be augmented to improve stability by considering and compensating time delays in position sensors and command actuators.

**Figure 5:**
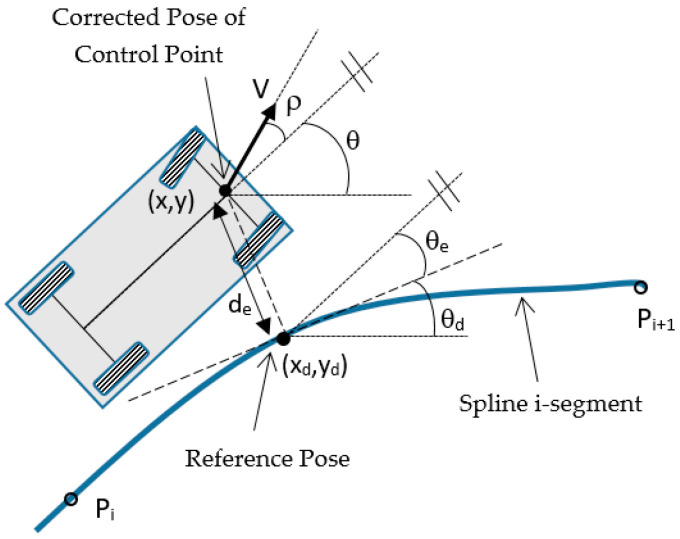
Variables involved in lateral control: reference pose *χ_d_* = (*x_d_*, *y_d_*, *θ_d_*) and tracking errors *ξ* = [*d_e_ θ_e_*]′.

We have implemented an LQR Optimal Controller for its stability characteristics and its easy tuning using cost functions. In order to linearize the non-linear kinematic model of the plant (Equation (1)), a convenient selection of the state vector of the controller is needed. The states are chosen to be the trajectory tracking errors *ξ* = [*d_e_ θ_e_*]′, defined as the distance from the control point to the closest point of the path (that we call reference pose *χ_d_* = (*x_d_*, *y_d_*, *θ_d_*)) and the difference between the vehicle heading and the tangent to the path in the reference point (see Figure 5), respectively.

#### 2.4.1. Reference Pose and Tracking Error Calculation

As the vehicle travels along the spline segments, the parameter *u*, that grows from 0 to 1 inside each segment, is a good way to detect segment changes. In this way, the current segment *i* and its polynomials *Y_i_*(*u*) and *X_i_*(*u*) coefficients are always known. To calculate the reference point (*x_d_, y_d_*), the value of *u = u_m_* that minimizes the Euclidean distance between the control point (*x, y*) and the trajectory must be calculated. Setting to zero the first derivative of the distance function, we obtain:

(12) $$A\cdot u_{m}^{5}+B\cdot u_{m}^{4}+C\cdot u_{m}^{3}+D\cdot u_{m}^{2}+E\cdot u_{m}^{}+F=0$$

with (for simplicity, the subindex *i* of the spline coefficients has been omitted):

(13) $$A=3d_{x}^{2}+3d_{y}^{2}\;B=5c_{x}d_{x}+5c_{y}d_{y}\;C=2c_{x}^{2}+4b_{x}d_{x}+2c_{y}^{2}+4b_{y}d_{y}\;D=3c_{x}b_{x}-3xd_{x}+3a_{x}d_{x}+3b_{y}c_{y}-3yd_{y}+3a_{y}d_{y}\;E=b_{x}^{2}-2xc_{x}+2a_{x}c_{x}+b_{y}^{2}-2yc_{y}+2a_{y}c_{y}\;F=a_{x}b_{x}-xb_{x}+a_{y}b_{y}-yb_{y}$$

The solution *u_m_* of the polynomial closest to the last value of *u* must be chosen, and when this solution overtakes 1, *i* must be incremented to change to a new spline segment. Therefore, the reference position is given by:

(14) $$\chi_{d}=\left(x_{d},y_{d},\theta_{d}\right)=\left(X_{i}\left(u_{m}\right),Y_{i}\left(u_{m}\right),atg\left(\frac{{Y^{\prime }}_{i}\left(u_{m}\right)}{{X^{\prime }}_{i}\left(u_{m}\right)}\right)\right)$$

The tracking errors are:

(15) $$\xi =\left[\begin{array}{c} d_{e}\\ \theta_{e} \end{array}\right]=\left[\begin{array}{c} \left(y-y_{d}\right)\cdot cos\left(\theta_{d}\right)-\left(x-x_{d}\right)\cdot sin\left(\theta_{d}\right)\\ \theta -\theta_{d} \end{array}\right]$$

#### 2.4.2. LQR Optimal Controller

Taking the tracking errors *ξ* as state vector, and the steering angle *ρ* as input, we can obtain the derivatives of the states from the kinematics of the vehicle (see Figure 3):

(16) $$\dot{\xi }\left(t\right)=\left[\begin{array}{c} \dot{d_{e}}\left(t\right)\\ \dot{\theta_{e}}\left(t\right) \end{array}\right]=\left[\begin{array}{c} V\cdot sin\left(\rho \left(t\right)+\theta_{e}\left(t\right)\right)\\ \frac{V\cdot sin\left(\rho \left(t\right)\right)}{L} \end{array}\right]$$

where *V* is the linear velocity of the vehicle, that is considered as a parameter for the design of the lateral controller. Assuming small errors and steering angles, we obtain the following linear state equation for the system:

(17) [de˙(t)θe˙(t)]=[0V00]·[de(t)θe(t)]+[VVL]·ρ(t)

and its discrete version, assuming the controller will work with sampling period *T_s_* is:

(18) [de(k+1)θe(k+1)]=[1V·Ts01]·[de(k)θe(k)]+[V·Ts+(V2·Ts22·L)Ts·VL]·ρ(k)

The control law is reduced to the following expression:

(19) $$\rho \left(k\right)=-K\cdot \xi \left(k\right)=-\left[\begin{array}{cc} K_{1} & K_{2} \end{array}\right]\cdot \left[\begin{array}{c} d_{e}\left(k\right)\\ \theta_{e}\left(k\right) \end{array}\right]$$

where *K* is calculated as a Linear Quadratic Regulator (LQR) so that it minimizes the following quadratic cost function:

(20) $$\begin{array}{c} \psi =\overset{N}{\underset{k=0}{\sum }}\xi^{T}\left(k\right)\cdot Q\cdot \xi \left(k\right)+\rho^{T}\left(k\right)\cdot R\cdot \rho \left(k\right)\\ =\overset{N}{\underset{k=0}{\sum }}\left[\begin{array}{cc} d_{e}\left(k\right) & \theta_{e}\left(k\right) \end{array}\right]\cdot \left[\begin{array}{cc} q_{11} & 0\\ 0 & q_{22} \end{array}\right]\cdot \left[\begin{array}{c} d_{e}\left(k\right)\\ \theta_{e}\left(k\right) \end{array}\right]+\rho \left(k\right)\cdot r\cdot \rho \left(k\right) \end{array}$$

The parameters *q*_11_, *q*_22_ and *r* allow an intuitive adjustment of the relative importance of minimizing the lateral error, the orientation error and the control effort, respectively. A good initial value for these parameters is the inverse of the quadratic maximum allowed deviation. The value of the feedback matrix *K* can be obtained from the Ricatti equation in stationary regime [52].

**Figure 6:**
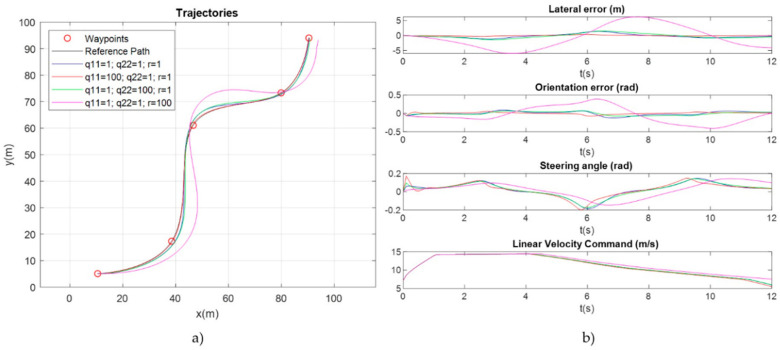
Influence of LQR controller parameters in the trajectory, the tracking errors and the control signal: (**a**) Resulting trajectories using (1) the same value for all the parameters (blue line), (2) a higher value for *q*_11_ (red line), (3) a higher value for *q*_22_ (green line) and (4) a higher value for *r* (magenta line); (**b**) tracking errors, control signal (steering angle) and linear velocity command provided by the linear velocity profiler.

Figure 6 shows some simulation experiments about the influence of *Q* and *R* matrixes (*q*_11_, *q*_22_ and *r* parameters) in the trajectory, the tracking errors and the actuation signal. Figure 6a shows the reference spline to follow in black color, and the resulting trajectories using: (1) the same value for all the parameters (blue line); (2) a higher value for *q*_11_ (red line); (3) a higher value for *q*_22_ (green line) and (4) a higher value for *r* (magenta line). As can be seen in Figure 6b, *q*_11_ and *q*_22_ minimize the lateral and orientation errors, respectively while a higher value of *r* results in a smoother signal control but with higher tracking errors. It can also be observed the linear velocity command provided by the linear velocity profiler along the path.

### 2.5. Delay Compensation

**Figure 7:**
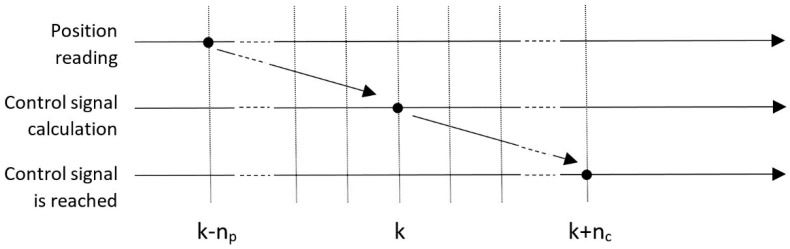
Delays involved in the control system.

The developed controller provides good results when the vehicle travels at slow speeds, so that the delays present in the robot’s sensors and actuators can be neglected. However, autonomous vehicles in urban environments sometimes must travel at high speeds (up to 50 km/h) and, in this situation, delays significantly destabilize the control system. There are two main delay sources to take into account in the control loop: the delay in the localization system readings, and the one introduced by the actuators. These delays are characterized as follows (see Figure 7):

- It is assumed that at the time *k* in which the control signal is calculated, the available position reading *χ_r_* corresponds to the vehicle position *n_p_* sampling periods before. If the vehicle travels at high speeds, this delay introduces a large error in the position used to calculate the control signal.
- It is assumed that the control signal calculated at time *k* will have effect on the vehicle after *n_c_* sampling periods. Obviously, at this time the vehicle will also have modified its position considerably if the speed is high.

**Figure 8:**
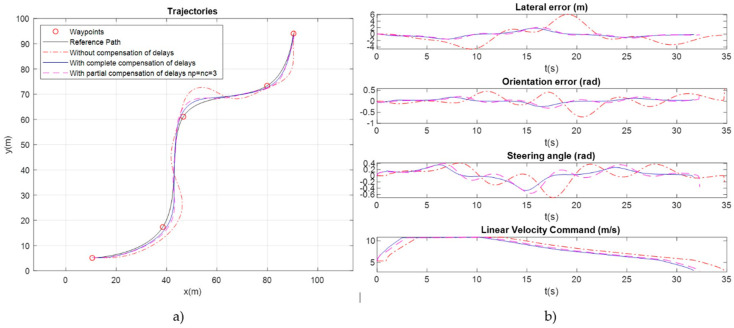
Effect of delays and their compensation: (**a**) resulting trajectories and (**b**) tracking errors and control signals. Delays of *n_p_ = n_c_* = 5 have been simulated in the sensors and actuators of the vehicle, and results are shown in the following cases: without compensation of delays (red dot-dashed line), with a partial compensation of the delays using *n_p_ = n_c_* = 3 in the compensator (magenta dashed line) and with a complete compensation of the delays (continuous blue line).

To test the effect of these delays, an example has been simulated with the vehicle traveling at a maximum forward speed of 14 m/s. The control algorithm is executed with sampling period T_s_ = 0.1 s, and 5 sampling periods of delay have been assumed in position reading and control actuation (*n_p_ = n_c_* = 5). Matrices *Q* and *R* have been experimentally adjusted to provide the best result, obtaining the trajectory and signals shown in red dot-dash line in Figure 8. As can be seen, the delays greatly destabilize the control system, with the lateral error approaching 6 m in the curve sections, which is not admissible for an urban autonomous vehicle.

For this reason, a delay compensation system is required (see Figure 2), which makes a prediction of the future position of the vehicle just when the actuation signal will take effect. For this, it is necessary to store in a buffer of size (*n_p_ + n_c_*) the last speed control signals sent to the actuators. In this way, at the sampling time *k*, the last known position *χ(k − n_p_*) will be integrated until the sampling instant (*k + n_c_*) using the vehicle model and the speeds stored in the buffer:

(21) $$\begin{array}{cccccc} for & n=k-n_{p} & to & n=k+n_{c} & & \chi \left(n\right)=\chi \left(n-1\right)+u\left(n-1\right)\cdot T_{s} \end{array}$$

being T_s_ the sampling period of the controller.

To check the operation of the compensation system, simulation results are shown in Figure 8. It has been assumed that the number of actual delays is *n_p_ = n_c_ =* 5. Results have been obtained with three different configurations, using the best adjustment of *Q* and R in each case: (1) without delay compensation (shown in red dot-dashed line), (2) compensating only part of the delay (shown in magenta dashed line), specifically *n_p_* = *n_c_* = 3 and (3) compensating the complete delay (shown in continuous blue line). Figure 8a shows the followed trajectories and Figure 8b the temporal evolution of the controller signals. As can be observed, lateral error is reduced from 6 m to less than 1 m using a compensation in which the number of real delays is known and compensated. Even if the number of delays cannot be known in practice and only part of them are compensated, the obtained improvement is relevant. In conclusion, for the proposed control algorithm to be viable in a vehicle traveling at high speeds in cities, it is essential to make a good estimation of the delays introduced by the measurement and actuation systems and compensate them conveniently.

## 3. ROS Implementation

The Robot Operating System (ROS) [14,53,54] is a meta-operating system with the philosophy to promote code reuse against the repetitive code work among the robot research community. As an open-source programing framework, ROS has been improved by hundreds of user-contributed ROS packages in the past few years [55].

One of the main resources of ROS is the “Navigation” stack [42,56], a set of packages that provides localization, mapping, planning and path following capabilities for autonomous robots. Within this stack, the “move_base” package [57] contains the local and global planners to achieve a navigation goal. However, these planners work with a single goal and optimize the trajectory using cost maps, that must be calculated from a previous map of the environment.

In order to achieve waypoint following, there are a set of ROS packages, such as “follow_waypoints” [43] or “yocs_waypoints_navi” [44] that basically take the waypoints in succession and pass them to “move_base” as independent goals. In addition, all the options available in ROS are designed for autonomous robots, but there are no specific waypoint tracking solutions for autonomous vehicles moving at higher speeds. In this case, as we saw in the previous section, it is necessary to compensate the existing delays in the control loop.

The controller proposed in this paper has been implemented as an ROS package called “Waypoint_Tracking_Controller” [45] [Supplementary Materials] in order to provide a real-time, robust, scalable and simple to use solution for ROS-based autonomous vehicles software. The ROS framework enables multiple programs being executed in parallel, what allows the control module to be easily incorporated into larger navigation architectures by means of message-passing communication.

The controller has been implemented in a single ROS node called “controller_node”, whose communication interface through ROS topics and ROS configuration parameters are detailed in Table 1. The node must subscribe to two input topics: “waypoints_input” is the sequence of waypoints to follow, and “absolute_pose” contains the current pose of the vehicle obtained by its localization system, both referred to the same absolute external frame. An optional input topic called “external_speed” allows the node to receive an external speed specification that can be combined with the velocity profiler command in different ways, depending on the value of the “speed_mode” parameter. This allows the speed of the vehicle to be adapted to the environmental and traffic conditions detected by an external perception module. The “min_dist” parameter adjusts a minimum distance between subsequent waypoints before interpolating them, so that some input waypoints can be discarded in a decimation process if they are close together.

As outputs, the “controller_node” publishes the interpolated path (“spline” topic) and the decimated waypoints used for interpolation (“points_spline” topic). The pose predicted by the delay compensator and the reference pose are also published in “predicted_pose” and “reference_pose” topics, respectively. These topics are useful for visualization purposes using ROS Rviz tool [58]. The main outputs of the controller are the control commands, published in “steer_cmd” and “speed_cmd” topics, and combined in the “cmd_vel” topic using ROS standards.

The operation of the controller is simply and intuitively adjusted using a small number of parameters shown in Table 1, and is described in detail in Section 2.

**Figure 9:**
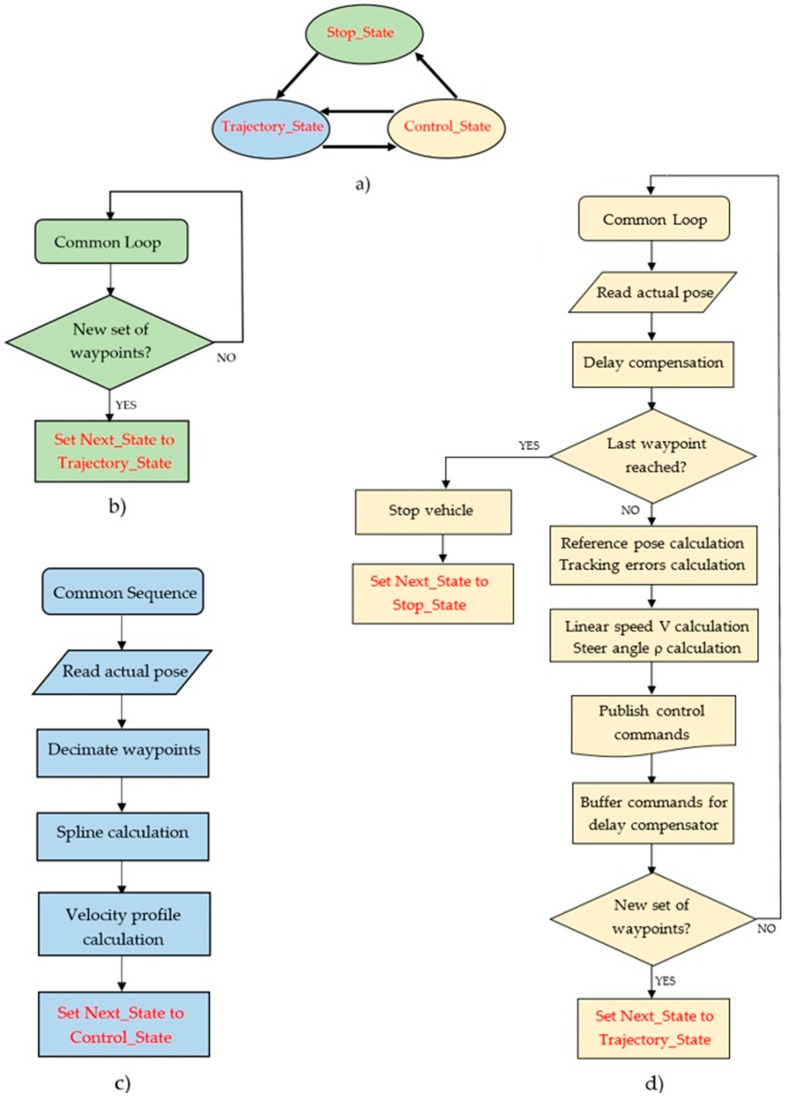
“Controller_node” operation: state machine is shown in (**a**), and the work flows of “Stop_State”, “Trajectory_State” and “Control_State” are shown in (**b**), (**c**) and (**d**), respectively.

Figure 9 shows the “controller_node” operation mode. A state machine (Figure 9a) controls the overall flow of the node. It starts in the “Stop_State” mode (Figure 9b), waiting to receive the sequence of waypoints, when it changes to “Trajectory_State” (Figure 9c). In this state the spline interpolator is executed. The waypoints provided as input do not have to be equidistant. However, if two consecutive waypoints are too close, the resulting spline segment may be so short that the vehicle overtakes it without any iteration of the controller, causing the algorithm to fail. To avoid this, a decimation process is performed to ensure a minimum distance between waypoints that can be adjusted through the “min_dist” parameter (whose default value is 5 m, appropriate for typical urban speeds). Once the waypoints have been filtered, the spline parameters and velocity profile are calculated. Finally, the “Control State” mode executes the control loop until the last waypoint is reached (when it returns to “Stop_State”) or any point in the waypoints list is modified (when it changes to “Trajectory_State” to recalculate the path). Within the control loop, the position provided by the vehicle localization system is corrected by the delay compensator before using it to calculate the reference position and tracking errors required by the optimal LQR controller.

The “Waypoint_Tracking_Controller” package is publicly available through GitHub [45]. In addition to the main control node, it includes some utilities, such as launcher files with default parameter configurations and some plugins to interface ROS bag files with Matlab software for visualization purposes.

## 4. Experimental Results and Discussion

This section shows the experimental results that validate the operation of the proposed controller. Firstly, the test bench used is presented, and then some ablation tests demonstrate the improvements introduced by the linear velocity profiler and delay compensator subsystems. Finally, a comparison with other state-of-art proposals is included.

### 4.1. Test Bench

To validate both the proposed controller and its implementation as an ROS package, the novel and hyper realistic CARLA Simulator [46,59] has been used. This is an open-source simulator for autonomous driving research that performs on highly realistic urban layouts, including a great variety of vehicle models, buildings, pedestrians, street signs and vehicle sensors that provide data that can be used to train multiple driving strategies in a variety of environmental conditions, including weather and time of day. As it provides very realistic sensory data for the development of perception systems, CARLA deals with a large community of researchers [60,61,62] that can benefit from a scalable and easy to adjust waypoint tracking control module. In addition, the “ROS-bridge” [63] provided by CARLA enables two-way communication between ROS nodes and the simulator, so that the information from the CARLA server is translated to ROS topics, and the messages sent between nodes in ROS get translated to commands to be applied in CARLA.

**Figure 10:**
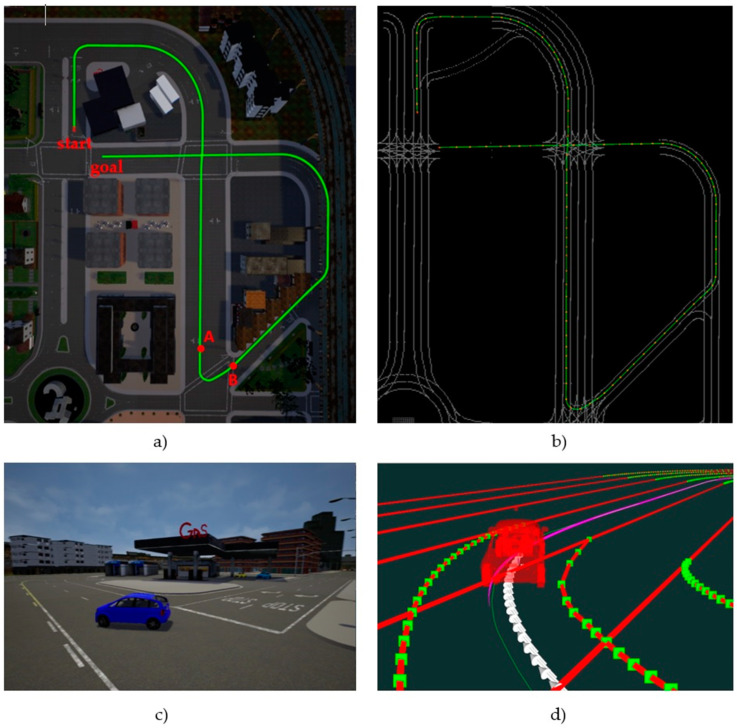
Test bench in the CARLA Simulator: (**a**) 610-m route in Town_03 used for the tests (AB segment is used to compare results in curves), (**b**) view of the route in Rviz Robot Operating System (ROS) visualizer, with the input waypoints shown as red points, (**c**) a closer view of vehicle model making the first turn in CARLA, (**d**) Rviz view of the same turn, with trajectory and reference pose markers for visualization.

Figure 10a shows a bird’s eye view of the route chosen for the controller tests. It is a 610-m route within Town03 CARLA scene including straight lanes and sharp curves. To define the path, a set of 122 waypoints along the route, from the start to the goal point, has been generated by CARLA as input for the controller, with an average distance of 5 m between them. These waypoints and the obtained interpolated spline can be seen in the Rviz view shown in Figure 10b. The vehicle used for the experiments is an Audi A2, whose model is available in CARLA. Figure 10c shows a closer view of the vehicle in CARLA simulator performing a curve, while Figure 10d shows the same view in Rviz, along with the path and the reference poses generated by the controller.

The main metric used to compare the different controllers shown in the following subsections is the Root Mean Square (RMS) value of lateral and orientation errors along the path, after 10 repetitions of each test. Since straight sections of the route mask the performance of the controllers on the more challenging curved sections, specific measurements for the sharped-curve between points A and B in Figure 10a will be included in all cases.

### 4.2. Ablation Tests

A set of tests were carried out to check the improvements introduced by the different modules of the proposed controller. As a baseline, the basic LQR controller was used, without incorporating the delay compensator or the velocity profiler. From this basic tracking control, we firstly incorporate the delay compensator, then only the velocity profiler and finally both together to obtain the complete controller.

**Figure 11:**
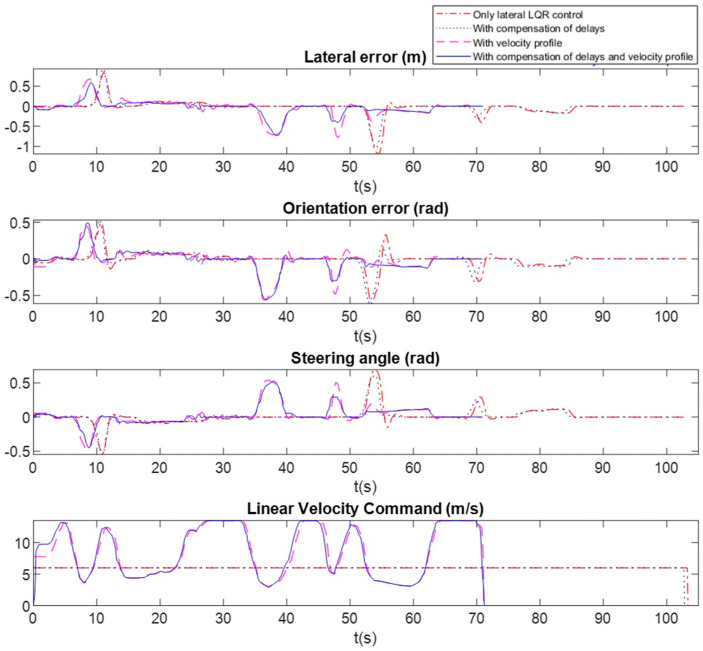
Ablation tests: using only the basic LQR lateral controller with 6 m/s constant speed (red dot-dashed line), using LQR lateral control + delay compensation (blue dotted line), using LQR lateral control + velocity profiler (with *V_MAX_* = 13.5 m/s) (magenta dashed line), using the complete controller with delay compensation and velocity profiler (blue line).

Figure 11 shows the lateral error, orientation error, steering angle and linear velocity along the path of Figure 10a in all cases. Table 2 shows the RMS value of errors during the complete route and in section AB (sharp curve), as well as the maximum, the average speed and the time to complete the route.

Results of the basic LQR controller are shown with red dot-dashed line in Figure 11. Since this baseline control does not incorporate the velocity profiler, the trajectory is made at constant speed. A speed of 6 m/s has been chosen so that the maximum lateral error in curves remains close to 1 m, and the route can be completed without the vehicle leaving the lane. In this case, the time to complete the route exceeds 100 s because a low velocity is maintained regardless of the path curvature.

The results after incorporating the delay compensator to the basic controller are shown with blue dotted line. After some experimental tests with the simulator sensors and actuators, a delay of *n_p_* = 10 and *n_p_* = 8 (using T_s_ = 0.1 s) were estimated for localization measurements and actuation, respectively. As can be seen, improvement due to the delay compensator at low speeds is not very significant, although a faster actuation is observed in curves, which slightly reduces tracking errors and allows the vehicle to complete the route in a shorter time.

A third test was carried out incorporating only the velocity profiler to the basic lateral control. This allows the controller to automatically adjust the velocity to the curvature of the path, so that higher speeds are chosen in straight sections of the path. The parameters of the velocity profiler have been set to *V_MAX_* = 13.5 m/s, *RC_MAX_* =20 m and *λ* = [0.5 0.3 0.1 0.1] (see Section 2.2). Results are shown with magenta dashed line in Figure 11. In this case the average speed along the route increases from 6 m/s to 8.65 m/s, but it decreases to values of up to 3 m/s in the areas with greater curvature. This allows the route to be completed in a shorter time, reducing the tracking errors (specially the lateral one) in curves. In the A–B section, the lateral error is reduced from 0.5 m to 0.35 m.

Finally, the complete proposed controller is tested, incorporating both the delay compensator and velocity profiler to the basic lateral control (results shown with blue continuous line). With respect to the previous test, it can be seen that the delay compensator further reduces tracking errors. The effect in this case is not so much noticed in the curves (since the speed is reduced in them) as in the straight sections of the route. Figure 11 shows that the oscillations on straight sections are considerably reduced, especially at the end of curves, when the vehicle increases its speed again.

The proposed controller completes the 610 m route in 71 s, with an average speed of 8.7 m/s that ranges from 3 m/s in sharp curves to 13.5 m/s in straight segments of the route. The average lateral error is 0.17 m, and it does not exceed 0.60 m in curves. All these values are very suitable for autonomous driving in urban environments.

### 4.3. Comparison with Other Proposals

In this subsection we show a comparison of our proposal with Pure Pursuit standard (usually used as benchmark to validate path tracking controllers) and our previous low level controller [47,64], an efficient and real-time algorithm (BCM-DO) based on the Beam Curvature Method that deals with dynamic obstacles, that treats the lane edges as static obstacles to perform a lane following controller.

**Figure 12:**
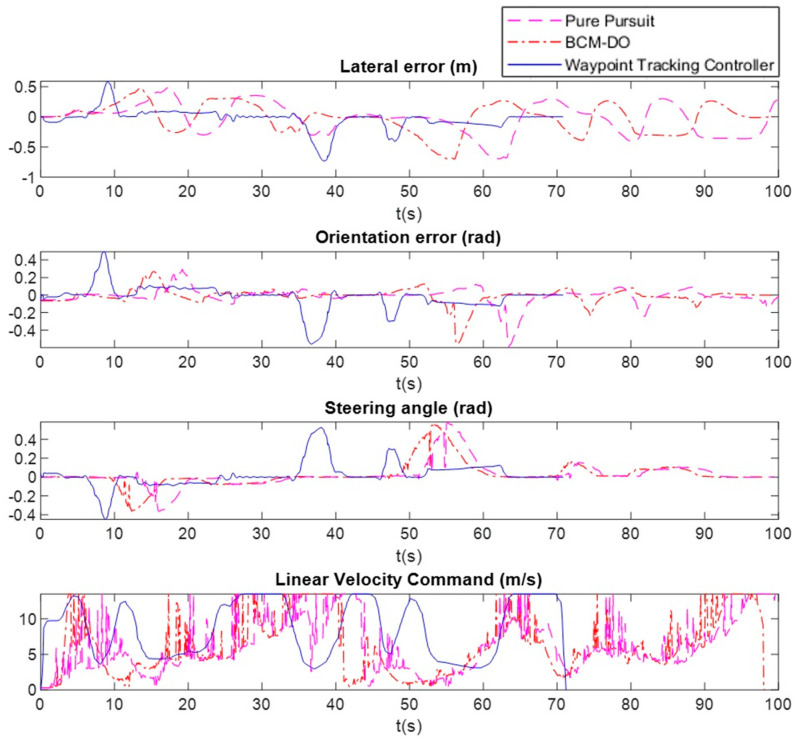
Comparison with other tracking controllers: Pure Pursuit (magenta dashed line), Beam Curvature Method that deals with dynamic obstacles (BCM-DO) (red dot-dashed line), proposed Waypoint Tracking Controller (blue continuous line).

These tests have been carried out on the same test bench as the ones in previous subsection, using the same metrics to compare the controllers. The common parameter for the three algorithms is the maximum linear velocity, set to *V_MAX_* = 13.5 m/s. Figure 12 and Table 3 show the temporal evolution and quantitative results, respectively.

As can be seen, our proposal maintains lower tracking errors with considerably higher average speed, completing the 610-m route almost half a minute earlier than the other two controllers. An important improvement of our proposal can be seen in the straight sections of the route, drastically reducing oscillations and achieving much lower average errors in the complete route. A much smoother speed profile is also observed than the ones generated by the two other control algorithms, avoiding abrupt changes in the control signals, which contributes to an easier controllability.

## 5. Conclusions

In this paper a controller is proposed for tracking paths defined by a set of input waypoints. Scalability, robustness, efficiency and real-time execution in urban environments, where vehicles drive at relatively high velocities, are the main requirements that have conditioned the design strategy.

The controller incorporates a cubic spline interpolator, which ensures continuity in the first and second derivatives obtaining a smooth trajectory. The result of this interpolator is a parametric path, instead of a larger set of points, which reduces memory requirements and computation times. The control algorithm is conceived entirely as a function of this parametric path.

The lateral controller is based on optimal control techniques, which ensure a robust and real-time operation with a simple and intuitive adjustment of their parameters. Two improvements have been incorporated to this basic lateral controller in order to adapt it to urban environments, where vehicles move at higher speeds than other types of autonomous robots. The first one is an automatic velocity profiler, which adjusts the linear speed according to the curvature of the path. This allows to select a maximum speed which is automatically reduced in the curved sections of the route as a function of their curvature. The second improvement is a delay compensator that corrects the instability due to delays in the measurement of vehicle localization and the actuation systems. This compensator makes a prediction of the vehicle pose just when control signals will have effect on it, using the kinematic model of the vehicle and the history of calculated control signals.

The proposed controller has been implemented as a package within ROS framework, one of the most widely used within the robotics research community. ROS does not yet provide any package that solves a control application for tracking waypoints at high speeds with smooth trajectories. The package, called Waypoint_Tracking_Controller, is available as open source in GitHub, and its integration in any navigation software is very simple due to its easy parameter adjustment, simplicity and reduced execution time, ensuring its real time implementation in different robotic platforms.

The controller and its ROS implementation have been validated using CARLA, one of the most complete and realistic vehicle simulators. Several tests have been conducted that demonstrate the correct performance of the controller in urban environments, at relatively high speeds. The LQR lateral controller is very stable, and its operation is clearly improved by the velocity profiler and the delay compensator.

The proposal has also been compared with one of the main standards used as benchmark in the validation of trajectory tracking systems, the Pure Pursuit algorithm. It has also been compared with the BCM-DO controller previously developed in our research group. It shows a much more stable behavior in straight sections of trajectories and lower tracking errors in curves, even at much higher speeds than the previous algorithms.

As future work, the controller will be integrated into the navigation architecture of the open-source electric vehicle available in our research group at the University of Alcalá in Spain [64].

## Figures and Tables

**Table 1:** List of subscribed topics, published topics and parameters for the “controller_node” node of the “Waypoint_Tracking_Controller” package.

**Subscribed Topics**			
**Topic Name**	**Message Type**		**Description**
waypoints_input	nav_msgs/Path		Sequence of input waypoints.
absolute_pose	nav_msgs/Odometry		Absolute position of the vehicle.
external_speed	std_msgs/Float64		External speed specification.
**Published Topics**			
**Topic Name**	**Message Type**		**Description**
Spline	nav_msgs/Path		Interpolated path for display purposes.
points_spline	visualization_msgs/Marker		Decimated waypoints for spline calculation, with minimum distance between points given by min_dist parameter. Useful for visualization purposes.
reference_pose	geometry_msgs/PoseStamped		Reference pose for control χ_d_ (see Figure 2).
predicted_pose	geometry_msgs/PoseStamped		Pose predicted by the delay compensator to calculate the reference pose.
steer_cmd	std_msgs/Float64		Front wheel steer angle command.
speed_cmd	std_msgs/Float64		Front wheel speed command.
cmd_vel	geometry_msgs/Twist		Linear and angular velocity command.
**Parameters**			
**Parameter Name**	**Type**	**Subsystem**	**Description**
n_max	Int	Spline Interpolator	Maximum number of input waypoints.
min_dist	double		Minimum distance between waypoints.
rc_max	double	Linear Velocity Profiler	Maximum value of the radius of curvature that reduces the linear velocity below v_max.
v_max	double		Maximum allowed linear velocity.
lambda_vector	List		Vector **λ** of normalized weights of the velocity profiler.
speed_mode	Int		Mode of combination of external speed specification and internal velocity profiler speed specification.
np	Int	Delay Compensator	Number of sampling periods estimated for the delay of the localization system.
nc	Int		Number of sampling periods estimated for the delay of the actuation system.
q11	double	LQR Lateral Controller	q11 parameter of the LQR lateral controller.
q22	double		q22 parameter of the LQR lateral controller.
r	double		r parameter of the LQR lateral controller.
Ts	double	All	Sampling period of the control system.

**Table 2:** Ablation tests results.

	Basic LQR Lateral Control	Lateral Control with Delay Compensation	Lateral Control with Speed Profiler	Lateral Control with Delay Compensation and Speed Profiler
Total lateral error (m) ^1^	0.1954	0.1680	0.2041	0.1733
AB Curve Lateral error (m) ^1^	0.5019	0.4395	0.3524	0.2924
Total Orientation error (rad) ^1^	0.1087	0.1074	0.1509	0.1126
AB Curve Orientation error (rad) ^1^	0.2285	0.2068	0.2268	0.2035
Average speed (m/s)	6	6	8.65	8.70
Maximum speed (m/s)	6	6	13.5	13.5
Time to complete the route (s)	103	102	71	70

^1^ RMS Value.

**Table 3:** Comparison with other tracking controllers.

	Pure Pursuit	BCM-DO	Proposed Waypoint Tracking Controller
Total lateral error (m) ^1^	0.2755	0.2842	0.1733
AB Curve Lateral error (m) ^1^	0.3179	0.3125	0.2924
Total Orientation error (rad) ^1^	0.1174	0.1055	0.1126
AB Curve Orientation error (rad) ^1^	0.1520	0.1471	0.2035
Average speed (m/s)	6.1	6.2	8.7
Maximum speed (m/s)	13.5	13.5	13.5
Time to complete the route (s)	100	98	70

^1^ RMS Value.

## Supplementary Materials

The video “A Waypoint Tracking Controller for Autonomous Road Vehicles using ROS Framework” (RobeSafe Research Group—University of Alcalá) is available online at https://www.youtube.com/playlist?list=PLh_tHW0c4RjnWn_UkWFcEjI9zmkVftpbc. The GitHub repositories with the implemented code are available online at https://github.com/RobeSafe-UAH/Waypoint_Tracking_Controller.
